# Looking to the future of organs-on-chips: interview with Professor John Wikswo

**DOI:** 10.4155/fsoa-2016-0085

**Published:** 2017-01-20

**Authors:** John P Wikswo

**Affiliations:** 1 Vanderbilt University, Nashville, TN 37235–1807, USA

**Keywords:** bioengineering, body-on-a-chip, drug development, microfluidics, organ-on-a-chip, systems biology, toxicology

## Abstract

**John Wikswo talks to Francesca Lake, Managing Editor:** John is the founding Director of the Vanderbilt Institute for Integrative Biosystems Research and Education (VIIBRE). He is also the Gordon A Cain University Professor; a B learned Professor of Living State Physics; and a Professor of Biomedical Engineering, Molecular Physiology and Biophysics, and Physics. John earned his PhD in physics at Stanford University (CA, USA). After serving as a Research Fellow in Cardiology at Stanford, he joined the Department of Physics and Astronomy at Vanderbilt University (TN, USA), where he went on to make the first measurement of the magnetic field of an isolated nerve. He founded VIIBRE at Vanderbilt in 2001 in order to foster and enhance interdisciplinary research in the biophysical sciences, bioengineering and medicine. VIIBRE efforts have led to the development of devices integral to organ-on-chip research. He is focusing on the neurovascular unit-on-a-chip, heart-on-a-chip, a missing organ microformulator, and microfluidic pumps and valves to control and analyze organs-on-chips.

## Q Can you tell us a little about your background & what led you into the organ-on-a-chip field?

At Vanderbilt I had a very strong research program that ranged from neuromagnetic measurements and cardiac biophysics to nondestructive testing and the study of corrosion in aging aircraft. In 2000, I decided to focus on applying microbioreactors, microfluidic sensors and controls to study cell biology, and that got me into the whole business of building devices to study single cells and small populations of cells. I worked with a large number of undergraduates, postdocs and staff and together we invented a new type of pump, which we called a rotary planar peristaltic micropump, and a valve called a rotary planar valve. In work on a variety of projects, we succeeded in refining the pumps and valves, and when the national organ-on-a-chip (OOAC) programs were announced by the Defense Advanced Research Projects Agency (DARPA), the NIH's National Center for Advancing Translational Sciences (NIH/NCATS), and the Defense Threat Reduction Agency (DTRA), I realized we had technology that was ideally suited to control OOAC perfusion and media recirculation and record the metabolic activity of OOACs. Essentially, I got fully into OOAC research in 2011 and have been working hard at it since then.

## Q What are you currently working on?

We have published a number of studies looking at the challenges, both technical and biological, in building OOACs and how to scale them properly when you want to study multiple interconnected OOACs. The organ we have the greatest experience with is the neurovascular unit, which recapitulates the blood–brain barrier (BBB) using human neurons, astrocytes, pericytes and microvascular endothelial cells. The third paper on our neurovascular unit/BBB unit has just been published in the *Journal of Neuroinflammation* [1] and we are using the device to study inflammation in the brain, the effects of stroke, and understand how these affect brain activation and signaling. We have just published a pair of papers in *Acta Biomaterialia* on a cardiac papillary muscle on a chip [2,3], which allows us to make quantitative biophysical measurements of the elastic, contractile, and electrical properties of cardiac muscle in ways that were not previously possible in other cardiac OOAC devices. As we can discuss in a minute, we are hard at work on creating a microformulator that allows us to control over time the mixture of media, nutrients, drugs, and toxins that are added to each individual well of a 96-well plate. We are also getting ready to publish papers on the pumps and valves I mentioned previously and the topologies of multiorgan perfusion and support. With my colleague John McLean and his group, we are making excellent progress on understanding OOAC metabolomics.

## Q What research questions will these projects hope to answer?

In addition to the obvious goal of using OOACs to guide drug development and identify adverse organ–organ outcomes, there are a number of other extremely exciting opportunities. At Vanderbilt and the University of Pittsburgh, the Environmental Protection Agency (EPA) is funding Shane Hutson, Lans Taylor, and our colleagues in collaborative OOAC research into developmental toxicology. I believe that OOACs will be critical in understanding how environmental toxins affect human development and physiology, for example, how aromatic hydrocarbons are implicated in endometriosis, or how other toxins to which the mother is exposed can adversely affect the development of the fetal brain or body. Equally important, there is a history in the field of physiology, from the late 1800s to the mid-20th century, of using isolated animal organs to understand physiological sensing and control. For today's physiology, I have introduced the useful concept of the hermeneutic circle of biology, wherein we cannot understand the whole of biology until we understand its parts and we cannot understand the parts of biology until we understand the whole [4,5]. Modern biology has taken us to the reductionist limit – the genome of individual humans and many other biological species. ‘Closing the circle’ involves using this fundamental information to work our way back to an understanding of the complete organism. One might do it computationally, for example, with systems biology models, but I expect that there will be serious computational obstacles in doing so. The alternative is to close the hermeneutic circle with synthetic biology, where we use engineered proteins, cells, tissues and now organs to reconstruct a homunculus – an *in vitro* model of a human at possibly one millionth the scale, so that we can ask fundamental questions about a simple model system that we have a hope of understanding, rather than a complete organism that is too complicated to fully comprehend. I am convinced that OOACs will play a central role in the next generation of integrative physiology studies. In this context, OOACs may be invaluable in our study of the mechanism of action of drugs, toxins, and chemical and biological warfare agents and their therapeutics. We now have access to transcriptomic, proteomic and metabolomic analyses that can track thousands of biological variables with time resolutions approaching a second. We need experimental systems that can detect with high sensitivity and rapidly control this breadth of signals – OOACs could fill that need.

## Q What are the biggest challenges you feel are facing OOAC research?

One of the great challenges in OOAC research is to keep a 3D tissue culture alive for an extended period of time. If you are working with induced pluripotent stem cells (iPSCs) or just assembling multiple cell types, it might take the cells 2 or 3 weeks or longer to reach a stage of development that is consistent with the properties you want. This is exemplified by formation of the BBB or differentiation of cardiomyocytes from a fetal to a more mature phenotype. The problem is keeping the organs alive long enough. Many people use gravity or pressure perfusion or rocking devices. I think the challenge lies in how to build a compact, low-cost and reliable way to meter fluids into the organs for at least a month. This would keep the cells alive and allow the different organs to interact correctly. I believe that it is critical to make devices that are small, easy to use, inexpensive and reliable, since for the OOAC technology to be fully successful it has to be widely accepted by not only the pharmaceutical markets, which are the target of a lot of the currently funded research, but also individual biologists and researchers trying to understand physiology, systems and developmental biology, and toxicology. In essence, from my point of view we need to focus on perfecting reliable, low-cost hardware that could support a variety of OOACs and organ–organ interactions.

There are a number of companies already producing OOAC devices for use by basic researchers and pharmaceutical companies, typically with single-pass perfusion – the devices are out there ready to be used. A new challenge arises when you want to have organs talk to each other, because you then have to start worrying about recirculation. The number of systems available that support recirculation is somewhat limited. The real question therefore becomes – are you trying to study individual organs or organ pairs, such as liver–heart or liver–brain? In the latter case it becomes more of a challenge.

## Q There has been a lot of discussion on the future of multiple organs on a chip, including the concept of a body on a chip. What are your thoughts on body on a chip?

I think body on a chip (BOAC) definitely needs to be done. The issue here is that you do not want to try to make a perfect microhuman – as I use the term homunculus. I basically argue that the homunculus is a ‘toy’ model and were you to make a perfect model, it would be too complicated to understand. Therefore, what you want to do is identify the minimal system needed to address a particular question. For example, if the question is the interaction of a drug metabolite with another organ, at the minimum you need, let us say, the liver, which metabolizes the drug, and the organ that is affected by the metabolite, such as the heart, brain or kidneys. If you were trying to do ADMET (absorption, distribution, metabolism, excretion and toxicity), you would need the appropriate organs. For instance, depending on how you want to get the drug into the system you may need skin, lung or gut. You then have to decide where the drug or toxin will be metabolized – the key places for that are primarily liver or kidney. Then, you have to ask where the metabolite is being stored. A candidate for that would be adipose tissue and in some cases muscle, as that is where you store a lot of glycogen. Finally, you have to ask about the end organ, which in my research is the brain. So, if you are interested in ADMET you need all those organs on the chip. However, if you are interested simply in maternal activation of inflammatory cytokines affecting the developing brain, you may not need more than the brain [6], or possibly the brain plus an immune system.

Overall, I think BOAC is going to be extremely important. The largest technical challenge is to get the fluid volumes within each organ and circulating between organs low enough that the compounds secreted by one organ are not diluted below a physiological threshold before they get to another organ. Many of the multiorgan systems currently in use miss that target by an order of magnitude or two.

As I said before, if you look at the history of physiology, the foundations of physiology were built with a combination of animal experiments and, more important, isolated organ experiments. Isolated organ experiments were being done from the late 1800s to the present day, but they started falling out of favor beginning in the 1950s when people could study isolated cells – for example, HeLa cells. Once you had an immortalized cell line in a laboratory you could study the cells themselves and physiology became much more cellular, and eventually much more molecular. What BOAC offers, which I think is an incredible opportunity, is the chance to start putting together organs in ways that allow you to study how organs interact as a physiological system. So, again, BOAC is going to be extremely good for ADMET, and for modeling physiological regulation and control – for example, how do you study serotonin homeostasis? You need a gut and you need a brain and a variety of other organs. I think BOAC is definitely a growth area for research.

## Q What other growth areas should we be focusing on in OOAC?

My group is hard at work developing a device we call a ‘microformulator’. Basically, this is a set of valves and pumps that allow one to mix very quickly – under automatic computer control and in very small volumes – solutions of media, drugs, toxins, nutrients and metabolites. This means that you can simulate, for example, the organs that you do not have in your BOAC or homunculus. The organs that are extremely important in physiology and are not necessarily included in many of the existing OOAC platforms are the organs that secrete hormones. You can run through a whole list of hormones that modulate the organs involved in ADMET, and in all of *in vitro* biology, whether it is cells grown on plastic, 3D tissue culture, printed organs or OOAC; however, people have been largely ignoring hormonal modulation. If I remember correctly, something like 56 of the top 100 drugs marketed worldwide have a molecular target that has circadian modulation [7] and the efficacy of a drug can vary between a factor of 2 or 10 over the course of a day owing to the modulation of hormones, nutrients and other biological signals. We are focusing on building a microformulator that will allow you to superimpose on anything from a 96-well plate to a Petri dish, or a 3D organoid to an OOAC, time-dependent hormonal regulation. I think that is going to introduce a whole new study in both OOAC pharmacology and in systems biology and physiology, because you will suddenly have temporal control of hormone levels in a manner that is very difficult to achieve using a standard pipetting robot as used in high-throughput screening.

We are currently funded by both a research contract with AstraZeneca and an NIH/NCATS small business innovative research (SBIR) grant through the CFD Research Corporation to build multichannel microformulators that can do anything from independently adjusting the concentrations of media components in every well in a 96-well plate to delivering and removing fluid from reservoirs on multiple OOACs. I think the concept of the microformulator is going to be extremely exciting. For example, it is going to allow you to explore the process of stem cell differentiation where you have to figure out what is the optimal sequence, combination, and time course of growth factors and nutrients needed to drive a fibroblast into iPSCs or other cells, and then to differentiate those iPSCs into their appropriate precursor and final differentiated population. Currently that requires a lot of manual effort or a large pipetting robot, and I think the microformulator will allow you to study this differentiation process with a great deal more speed and parallelism and lower cost than is currently possible.

## Q How far away from completion is this research?

We have delivered a version one of the microformulator to AstraZeneca that is currently living in one of their incubators – it is a very complicated widget. We are close to delivering two of the second version, which is going to be much smaller and easier to maintain, and I would expect that within a year we will be producing preproduction prototypes for our own laboratory and those of our collaborators.

## Q VIIBRE has a large focus on enhancing interdisciplinary work. What advice would you give others looking to improve their interdisciplinary collaborations?

Much of science has basically evolved into a large number of people drilling very deeply into very specific areas, so there is a silo mentality where a person might spend much of his or her career studying a single protein family, for example. Interdisciplinary research requires people that can speak at least two languages – their native language that they are deeply interested or skilled in, whether it be biology or chemistry, among others, and one of the other languages, whether it is biomedical engineering or analytical chemistry, amongst others. Essentially, you have to be able to find people that can span multiple disciplines. I think at Vanderbilt we are fortunate because we have a large number of people that can clearly understand multiple languages. Once you have the right people, you have to be able to identify problems that interest all parties, or as many of the parties as you can. Finally, what you have to do is have an institutional recognition that interdisciplinary research is very important, yet challenging and quite different from the standard drill-deep mentality. If you look at what I call ‘intellectual phase space’, which basically describes each dimension as an area of knowledge or thinking, whether it is biological physics or engineering or another discipline, people have drilled so far down into those areas that the distance to the frontier of any other individual field is very large. But, if you look at the distance to the frontier between two fields, it can be closer than you might expect! Overall, I think the challenge is to identify meaningful problems that are not adequately explained by an individual discipline and to figure out which ones are important. When you identify them you suddenly realize that you can make contributions without having to go to the absolute frontier of any single discipline.

You can read more about John's phase space concept and his homunculus work in his TEDx talk [8].

## Q If you had unlimited funding, what would you do with it to further OOAC research?

I would start an intensive program to characterize the response of different organs: animal organs *in vitro*, animal cells in culture, human cells in culture and human cells used to create OOACs. What I would try to do is launch a program of intense characterization of the organs to try to understand the extent to which the *in vitro* cells on plastic and OOACs in both humans and animals replicate real physiology. There is a paper I just read by a friend of mine, Jim Stevens at Eli Lilly, whose group did a weighted gene co-expression network analysis studying the comparison of *in vivo* rat liver, *in vivo* mouse liver and rat primary hepatocytes grown on a dish [9]. They took as their gold standard the rat liver *in vivo*, and found that the best model of the rat liver *in vivo* was a mouse liver *in vivo*, and that the rat primary hepatocytes growing quietly on a layer of collagen on a plate in the laboratory looked more like rat liver that had been exposed to an extremely toxic drug. The challenge in growing primary hepatocytes in a dish is that the trauma of being removed from a rat and grown on plastic without the right cellular neighbors and the correct media is about as drastic as the trauma of the intact rat being exposed to a highly toxic drug. I think the premise is, although it has not yet been proven universally, that OOAC does a better job of recapitulating human physiology than does biology on plastic. I think that is probably a very valid hypothesis but it has to be tested rigorously. The way to test it is by doing extensive proteomics, metabolomics and transcriptomics on not only the OOAC but the model systems you are comparing it with and decide the extent to which your *in vitro* models actually replicate *in vivo* physiology. I am involved in the DARPA Rapid Threat Assessment (RTA) program that is developing the ability to determine the mechanism of action of a drug within 30 days, rather than the typical 10 years it can take to identify some of the off-target effects. The Vanderbilt RTA group, led by Richard Caprioli, is developing analytical techniques and network analyses for proteomics, metabolomics, phosphoproteomics, transcriptomics, and end point assays with high spatiotemporal resolution. I think that our entire RTA analytics platform applied to OOAC would be absolutely the best way to fully understand physiology, pharmacology, and toxicology. The approach would be even stronger were we to suppress specific genes or apply challenge compounds as we refine and validate the mechanism of action.
